# Fertility Sparing Surgery and Borderline Ovarian Tumours

**DOI:** 10.3390/cancers14061485

**Published:** 2022-03-14

**Authors:** Lorraine S. Kasaven, Mehar Chawla, Benjamin P. Jones, Maya Al-Memar, Nicolas Galazis, Yousra Ahmed-Salim, Mona El-Bahrawy, Stuart Lavery, Srdjan Saso, Joseph Yazbek

**Affiliations:** 1 Department of Cancer and Surgery, Imperial College London, South Kensington Campus, London SW7 2AZ, UK; 2 Department of Cutrale Perioperative & Ageing Group, Imperial College London, South Kensington Campus, London SW7 2AZ, UK; 3 Department of Obstetrics and Gynaecology, West Middlesex University Hospital, Chelsea and Westminister NHS Foundation Trust, London TW7 6AF, UK; mehar.chawla@nhs.net (M.C.); benjamin.jones@nhs.net (B.P.J.); 4 Department of Obstetrics and Gynaecology, Queen Charlotte’s and Chelsea Hospital, Imperial College NHS Trust, London W12 0HS, UK; maya.almemar@nhs.net; 5 Department of Obstetrics and Gynaecology, Imperial College NHS Trust, London W12 0HS, UK; nicolas.galazis@nhs.net (N.G.); Yousra.ahmed-salim@nhs.net (Y.A.-S.); 6 Department of Metabolism, Digestion and Reproduction, Imperial College London, Hammersmith Hospital, London W12 0HS, UK; m.elbahrawy@imperial.ac.uk; 7 Department of Reproductive Medicine, Hammersmith Hospital, Imperial College NHS Trust, London W12 0HS, UK; stuart.lavery@nhs.net; 8 Department of Gynaecological Oncology, Queen Charlotte’s and Chelsea Hospital, Imperial College NHS Trust, London W12 0HS, UK; srdjan.saso01@imperial.ac.uk (S.S.); joseph.yazbek@nhs.net (J.Y.)

**Keywords:** fertility-sparing surgery, borderline ovarian tumour, ultrasound guided laparoscopic ovarian wedge resection, ovarian cancer, intra-operative ultrasound

## Abstract

**Simple Summary:**

Fertility-sparing surgery (FSS) is now a widely acceptable treatment for the management of Borderline Ovarian Tumours (BOTs) in women of reproductive age. However, many clinicians face the dilemma of balancing the risks of disease recurrence with progression to lethal malignancy whilst preserving fertility, in the absence of clear standardized guidelines. The aim of this study was to evaluate the oncological outcomes in women who underwent FSS for the management of primary, or recurrent presentation of BOTs, to provide clinicians with further evidence of the safety and feasibility of FSS. Oncological outcomes following a novel method of FSS in the form of laparoscopic ultrasound guided ovarian wedge resection has also been introduced, which has the potential to change the way BOTs are managed in the future in women of reproductive age.

**Abstract:**

To determine the oncological outcomes following fertility-sparing surgery (FSS) for the management of Borderline Ovarian Tumours (BOTs). A retrospective analysis of participants diagnosed with BOTs between January 2004 and December 2020 at the West London Gynaecological Oncology Centre was conducted. A total of 172 women were diagnosed; 52.3% (90/172) underwent FSS and 47.7% (82/172) non-FSS. The overall recurrence rate of disease was 16.9% (29/172), of which 79.3% (23/29) presented as the recurrence of serous or sero-mucinous BOTs and 20.7% (6/29) as low-grade serous carcinoma (LGSC). In the FSS group, the recurrence rate of BOTs was 25.6% (23/90) presenting a median 44.0 (interquartile range (IQR) 41.5) months, of which there were no episodes of recurrence presenting as LGSC reported. In the non-FSS group, all recurrences of disease presented as LGSC, with a rate of 7.7% (6/78), following a median of 47.5 months (IQR 47.8). A significant difference between the type of surgery performed (FSS v Non-FSS) and the association with recurrence of BOT was observed (Pearson Chi-Square: *p* = 0.000; x = 20.613). Twelve women underwent ultrasound-guided ovarian wedge resection (UGOWR) as a novel method of FSS. Recurrence of BOT was not significantly associated with the type of FSS performed (Pearson Chi- Square: x = 3.166, *p* = 0.379). Non-FSS is associated with negative oncological outcomes compared to FSS, as evidenced by the higher rate of recurrence of LGSC. This may be attributed to the indefinite long-term follow up with ultrasound surveillance all FSS women undergo, enabling earlier detection and treatment of recurrences.

## 1. Introduction

Globally, epithelial ovarian cancers account for the second-most-common cause of death from a gynaecological cancer [1,2]. Borderline Ovarian Tumours (BOTs) comprise of a separate entity of non-invasive epithelial neoplasms, with a recognised, albeit uncertain potential for malignancy, as acknowledged by the International Federation of Gynaecology and Obstetrics (FIGO) [3]. As such, BOTs account for 15% of all epithelial ovarian malignancies (EOMs) [4]. The incidence is low, with European series reporting 4.8:100,000 new cases per year [5]. Histologically, they show more complex architecture, cellular crowding, proliferative activity, and variable nuclear atypia when compared to benign ovarian tumours of the same histological subtype [6]. The absence of stromal invasion differentiates BOTs from ovarian carcinomas [7].

BOTs can be of several histological subtypes, of which 53–65% are of the serous (sBOT) and 32% of the mucinous (mBOT) type. Less than 5% include the sub-types: clear cell, endometrioid and Brenner tumours [3,4,8]. BOTs are staged according to the FIGO staging; however, unlike ovarian carcinoma, at least 70–80% of cases are diagnosed at an earlier stage I [8]. Presentation of disease at stage II and III occurs in nearly 21% [9], with <1% of women diagnosed at stage IV [3]. Approximately 16–30% are asymptomatic at diagnosis [10]. The prognosis of BOTs is excellent [11], with the five-year survival rate for women with FIGO stages I–III described as 95–99.7% and stage IV disease as 77.1% [12].

A third of patients diagnosed with BOTs are <40 years of age and comparatively present 10 years younger than women with ovarian carcinoma (45 versus 55 years old respectively) [13,14,15]. Given that the majority of women are of reproductive age at diagnosis, the demand for fertility preservation in the form of conservative surgery is crucial. For women who no longer wish to preserve fertility, radical surgery, also referred to as complete debulking, aims to provide optimal management of the disease. This includes abdominopelvic exploration, peritoneal washings, and excision of the macroscopic tumour, followed by bilateral salpingo-oophorectomy (BSO), omentectomy, and peritoneal biopsy [16]. In women who wish to preserve their reproductive potential however, fertility sparing surgery (FSS) is the treatment option of choice where feasible. This includes resection of the BOT through conservative surgical procedures including ovarian cystectomy, unilateral salpingo-oophorectomy (USO), and more recently a novel technique of ultrasound-guided ovarian wedge resection (UGOWR) [16,17]. In women with bilateral ovarian involvement, a USO and ovarian cystectomy, or bilateral ovarian cystectomy, may also be indicated [6]. In such instances where peritoneal washing, omentectomy, and peritoneal biopsy is performed, women would be considered having undergone a complete staging of disease.

The predominant risk associated with FSS however, includes the recurrence of disease, which is reported to be between 5–34% [2,18]. Evidence also suggests recurrence is 2–4 fold higher when compared to radical surgery [19]. This is exemplified in various studies whereby 18–36% of women developed a recurrence following ovarian cystectomy, compared to 0–5.7% following radical surgery [20,21]. For this reason, individualised and carefully planned surgical management is imperative, to ensure the risks of disease recurrence are balanced with fertility preservation and restoration of the ovarian reserve. Post-operative surveillance with ultrasound monitoring for sonographic appearances is therefore essential in early detection of disease recurrence.

Although FSS is widely acceptable, for many clinicians, the dilemma of balancing the risks of disease recurrence with progression to lethal malignancy, whilst preserving fertility, remains challenging. The lack of clear standardized guidelines regarding the optimal method of conservative treatment is a major drawback, often resulting in a few women who are either inadequately, or overly, treated for their pathology. Therefore, demand for further evidence regarding both the oncological and subsequent fertility outcomes is required. The objective of this study was to evaluate such outcomes in women who underwent FSS for the management of BOTs. This included assessing the recurrence rate of non-invasive or invasive disease following surgery and reporting the number of women who achieved pregnancy, either spontaneously or through in vitro fertilisation (IVF) and the associated pregnancy outcomes.

## 2. Materials and Methods

This was a retrospective cohort study of all patients diagnosed with BOTs between 1 January 2004 and 31 December 2020 at the West London Gynaecological Cancer Centre, Imperial College Healthcare NHS Trust, London, United Kingdom.

Surgical management of BOTs were defined as radical or conservative FSS. The criteria for the latter included the desire to preserve fertility and the ability to comply with regular follow-up and surveillance monitoring. Other factors when considering management included the diagnostic ultrasound staging of disease (extra-ovarian lesions), whether presentation of disease was primary or a recurrence, the complex anatomy from previous surgery, the intra-operative findings, the completion of the family and the age of the woman.

Conservative surgery involved preservation of the uterus and at least part of one ovary. As such, unilateral or bilateral ovarian cystectomy, UGOWR, and USO were considered FSS. UGOWR involves the simultaneous use of transrectal ultrasound imaging during laparoscopy to aid the surgeon when visualizing ovarian tissue and identifying BOT lesions. Occasionally, FSS techniques were combined, i.e., USO and contralateral ovarian cystectomy. Uterine preservation was also considered FSS, if the aim was to achieve pregnancy via IVF or through a donor egg. Radical surgery, also referred to as complete debulking, included procedures whereby participants underwent at least a BSO, peritoneal washings, biopsies and omentectomy, and a total abdominal/laparoscopic hysterectomy for serous pathology. For mucinous BOTs, an appendicectomy was performed. In the context of sero-mucinous pathology, the decision for appendicectomy and/or lymphadenectomy was individualised. Subgroup analysis of the FSS group enabled comparisons to be made between the following groups: USO, ovarian cystectomy, UGOWR, and other fertility-sparing surgery whereby combined procedures were undertaken.

The 2014 FIGO classification was used to determine the stage of disease, as per surgical and histopathological findings discussed during subsequent gynaecological multidisciplinary meetings [22]. All women who underwent FSS were indefinitely followed up in our tertiary centre, whereby a centralised ovarian clinic was established. Follow-up occurs every three months for the first two years, six-monthly for the next three to five years and annually thereafter. Women who underwent non-FSS were often discharged back to their local hospital for follow-up, which occurs at six monthly intervals for three years and annually thereafter during year four and five. If a recurrence of disease was suspected, the woman was referred back to our centre for further management. All clinical, surgical, and histopathological information was collected using the medical records.

### Statistical Analysis

All statistical analyses were performed using SPSS version 24 software (SPSS, Chicago, IL, USA). Descriptive statistics were used to characterise patient demographics. The Kruskal-Wallis test was used to compare histological subgroups. The Pearson Chi Square and Fischer’s Exact Tests were carried out to determine the significance of recurrence of disease between sub-groups. Statistical significance was determined by a *p* value < 0.05. 

## 3. Results

Three histopathological subtypes of BOT were diagnosed: 69.8% (120/172) serous, 25% (43/172) mucinous, and 5.2% (9/43) sero-mucinous. The median age of presentation was 42 (IQR 24), 31 (IQR 26.5), and 35 (IQR 15) years, respectively (Independent Samples Kruskal–Wallis Test *p* = 0.040). The longest duration of follow-up was 9.3 years (112 months). There were no cases accounting for clear cell, endometrioid or Brenner tumour histopathological subtypes within the study population. The median duration of follow-up by our unit was 37 months (IQR 49.5) amongst the FSS group, compared to 14.5 months (IQR 34.8) in the non-FSS group.

Table 1 compares the ultrasonographic features at diagnosis between the FSS and non-FSS group.

Table 2 compares the histopathological features at primary surgery between the FSS and non-FSS groups.

Table 3 reports the various surgical procedures performed for the initial management of BOTs. Table S1 can be found in the Supplementary Material and summarises all FSS, whereby combined procedures were undertaken, referred to as “Other FSS”.

Table 4 demonstrates the recurrence rate (%) of BOTs from each type of surgical procedure performed.

Table 5 reports the statistical significance of recurrence of BOTs, when types of FSS are compared.

Table 6 compares various variables amongst women with non-invasive recurrences to those with malignant transformation of disease.

All reproductive outcomes are displayed in Table 7. The number of women who expressed a desire to conceive post operatively amongst the serous, mucinous, and sero-mucinous histological subtypes were 33 (27.5%), 20 (46.5%), and 4 (44.4%), respectively. The numbers of women considered of reproductive age (≤45 years) were 68 (56.7%), 31 (72.1%), and 7 (77.8%) amongst the same subgroups, respectively.

Table 8 summarises the surgical and reproductive outcomes from women who underwent UGOWR.

### 3.1. Recurrence of BOTs

The overall recurrence rate of disease was 16.9% (29/172), of which 79.3% (23/29) presented as serous or sero-mucinous BOTs and 20.7% (6/29) as low-grade serous carcinoma (LGSC). In the FSS group, the recurrence rate of BOTs was 25.6% (23/90) and diagnosed a median of 44.0 (IQR 41.5) months post-operatively. No recurrences of LGSC were reported in this group. In the non-FSS group, all recurrences of disease presented as LGSC, with a rate of 7.7% (6/78) and a median of 47.5 months (IQR 47.8) post-operatively. Recurrences of BOTs occurred within the subgroups of serous 22.5% (27/120) and sero-mucinous 22.2% (2/9) pathology only. Thus, statistical analysis for recurrence of BOT was performed for the serous group only, as the numbers were too small in the mucinous and sero-mucinous groups. There was a statistically significant difference between whether FSS or non FSS was performed and association with recurrence of BOT (Pearson Chi- Square: *p* = 0.000; x^2^ = 20.613). However, there was no significant difference between the type of FSS performed and recurrence of disease (Pearson Chi- Square: *p* = 0.379; x^2^ = 3.166.)

#### 3.1.1. Serous Borderline Ovarian Tumours

Within the serous group, the overall recurrence rate was 22.5% (27/120), with a median of 44.0 (IQR 48.0) months following surgery. From these recurrences, 22.2% (6/27) presented as LGSC, all of which occurred in women who had undergone non-FSS. Three of these women subsequently underwent secondary complete debulking and adjuvant chemotherapy and have not since developed further recurrences. Of the remaining three, two presented with peritoneal recurrences (one with LGSC and the other non-invasive implants) too small to operate on and was therefore treated with chemotherapy only. Both remained under follow-up within our centre, with no further recurrences reported. The third woman was referred for a first recurrence of disease 93 months following surgery. She underwent secondary debulking but developed a second recurrence 53 months later, for which she is currently being managed by palliative radiotherapy. The survival rate at the time of writing was 100% for all recurrences presenting as LGSC.

The remaining recurrences of 77.8% (21/27) presented as sBOT following FSS surgery with a median of 44.0 (IQR 44.0) months. From this subgroup, 28.6% (6/21) underwent complete debulking for treatment of first recurrence and have since developed no further recurrences. Next, 71.4% (15/21) underwent further FSS for management of their first recurrence, in which 33.3% (7/21) women have since developed a second recurrence of sBOT, with a median of 27.0 (IQR 15.0) months following surgery. One woman (4.8%) developed a third recurrence of sBOT eight months after surgery. At each stage of disease, she underwent FSS initially with ovarian cystectomy, followed by UGOWR after the first, second, and third recurrence.

#### 3.1.2. Sero-Mucinous Borderline Ovarian Tumours

In the sero-mucinous group two women recurred following FSS with non-invasive implants of BOT. The first initially underwent laparoscopic ovarian cystectomy performed at a different unit. She was referred to our centre 19 months after surgery whilst pregnant with recurrence. During an emergency caesarean section, the lesion was de-roofed and drained. Histology confirmed a smBOT, for which she then underwent laparoscopic USO to treat. She continues her follow-up at our unit with no further recurrences reported.

The second woman initially underwent laparoscopic USO and contralateral ovarian biopsy, in which histology confirmed non-invasive implants with stage IIIa disease. A first recurrence was diagnosed 47 months later, which was treated with UGOWR. Histology confirmed a serous cystadenofibroma with features of borderline changes within <10% of the lesion resected. Although current guidelines would define this as benign, and not a BOT, in view of the initial staging of disease, she was classified as a recurrence. Her second recurrence of smBOT developed 11 months after, for which she declined surgery and opted for conservative management. She has since attended 18 clinic appointments over the course of 83 months from her first presentation of BOT, with stable appearances of disease recurrence.

## 4. Discussion

Data from this study confirm that women of reproductive age undergoing surgical management of BOT can be managed safely when FSS methods are implemented. Whilst the literature widely reports that oncological outcomes are favourable, there is a lack of clear international guidelines to advise on the optimal FSS approaches. This study not only includes one of the largest sample sizes but also a long duration of consistent follow-up (nine years), compared to previously published retrospective studies of less than five years.

Evidence strongly suggests that FSS compared to non-FSS is an independent risk factor for recurrence of disease through multivariate analysis [23,24]. The recurrence rate of BOTs following FSS is between 5–34% [2,18], approximately five-fold higher when compared to recurrence after radical surgery quoted as 3.2–7% [25,26]. This is exemplified from the observed differences in our own cohort of all pathology (FSS 25.6% v. non-FSS 0% of non-invasive recurrences). The risk of progression to invasive carcinoma following conservative surgery however is 2#x2013;3% [27]. Within our study there were no recurrences of LGSC or invasive implants reported in the FSS group, whereas 7.7% (6/78) presented as LGSC in the non-FSS group. Although higher rates of malignant transformation (30%) have been reported, for example in the multi-centre ROBOT study [28,29], it was not specified whether the malignancy occurred in the FSS or the non-FSS group. Furthermore, it has been suggested that following a five year follow-up period, at least a third of all recurrences present as invasive disease [9]. The overall rate of malignant transformation from our study, however, was 20.7% (6/29), which is much lower. Additionally, all invasive cases presented in this study were of LGSC, whereas in the ROBOT, half the invasive relapses presented as LGSC and the remaining as high-grade serous carcinoma [28,29].

Current practice amongst many European countries is such that women who have been treated with radical surgery are discharged after five years of follow-up. In a study of 1143 Danish women, it was deduced that long-term follow-up is not necessary in the management of stage 1a BOT, in the absence of residual disease or microinvasion [30]. This was attributed to the low recurrence rate of BOT, identified as 3.7% (42/1143), in a cohort of women with predominantly (88%) FIGO stage 1 disease [30]. However, only 16% of women in the study underwent FSS [30], compared to 45.3% of our own cohort. Considering that FSS is associated with disease recurrence, the low numbers of women undergoing FSS in the Danish study may account for the overall low recurrence rate reported. Furthermore, given that the primary treatment of BOT in Denmark is radical surgery, it can be assumed that the reported high rate of malignant transformation observed (40.5%, 17/42), was in those who underwent radical surgery [30]. Evidently, our findings support that the prognosis of women treated with radical surgery is worse than those managed with FSS. Hence, this group of women should be managed similarly to those who undergo FSS, by means of long-term follow-up and surveillance monitoring to prevent malignant recurrences developing.

One could argue various factors may be attributed to the increased rate of malignant transformation observed in the non-FSS group, including consideration for FIGO staging, age, or the histological subtype at diagnosis. Table 6 demonstrates that type of surgery, e.g., FSS vs. non-FSS (*p* = 0.002; x^2^ = 9.9) and histological diagnosis (*p* = 0.000; x^2^ = 21.55) were both significantly associated with malignant transformation, whereas age (*p* = 0.99; x^2^ = 7.0) was not. It is therefore important for clinicians to also consider these factors when planning surgical management of disease.

The rate of death because of progression to malignancy following FSS is reported as 0–3% [21,31,32]. Thus, our findings of no reported incidences of death following FSS are lower. Furthermore, despite an increased risk of recurrence with FSS, it does not appear to worsen the survival rate of patients and should therefore remain the first line of treatment in women who wish to conceive.

Evidence regarding the relationship between the risk of recurrence and the method of FSS is somewhat equivocal. Various studies suggest there is no significant difference in disease recurrence with the method of FSS [23,32,33]. A recent meta-analysis suggested, however, that disease relapse is highest when ovarian cystectomy for unilateral serous BOTs are performed, with a recurrence rate of 25.3% compared to 12.5% with USO (*p* = 0.0001) [34]. When deciding which FSS to offer women, it may be important to consider the histological subtype of the tumour. For example, knowledge that a third of serous BOTs present bilaterally may suggest that USO does not protect against contralateral ovarian recurrences. This was demonstrated in a study whereby 22% of women developed contralateral disease following USO [2]. As such, future ovarian surgery will result in less healthy ovarian tissue, increased risk of adhesions, and tissue damage impacting future fertility outcomes. In women requiring multiple surgeries for recurrences, the risk of premature ovarian failure has also been observed within 12 months of surgery for a second recurrence [2]. Fertility, therefore, appears to be more influenced by the frequency of surgical intervention rather than the type of FSS. Therefore, decisions should reflect this when managing women of reproductive age.

### 4.1. Ultrasound-Guided Ovarian Wedge Resection

Within our tertiary Gynaecological Oncology Centre, a specialist expert ovarian ultrasound clinic was dedicated to the diagnosis and management of BOTs. With improved technology in ultrasound quality and clinician expertise, we advocate that intraovarian deposits of ovarian borderline disease of increasingly smaller size can be detected earlier. Such deposits, too small for the naked eye, may be missed during therapeutic laparoscopic staging or resection of disease, resulting in patients being upstaged, thus undergoing oophorectomy to ensure complete resection. Alternatively, the plan may be to continue surveillance monitoring until the disease can be visualised laparoscopically, and only then surgically resected. As such, in the absence of expert scanning resources, the clinician may be inclined to over- or under-treat pathology.

Intra-operative ultrasound has been described as a novel adjunct to FSS, assisting the resection of small ovarian lesions and optimising the chances of complete resection of disease, whilst preserving maximum ovarian tissue [16]. When performing FSS on smaller borderline ovarian deposits, disruption to healthy ovarian follicles has been observed, as demonstrated by lower relative follicle densities during cystectomy for endometriomas [35]. Furthermore, this method has the potential to enhance intra-operative diagnostic accuracy when delineating pathology within the ovary [36]. In the context of sBOTs, one third of deposits present bilaterally [16]. Although previous practice suggested obtaining an ovarian biopsy of the contralateral ovary, this is no longer advised in the absence of visible disease [8]. Therefore, the use of detailed intra-operative ultrasound assessment of the contralateral ovary may assist the surgical staging of disease and the earlier diagnosis of recurrence. Simultaneously, it may prevent injury to healthy follicles sustained from the disruption to the ovarian tissue when a biopsy is taken and thus, the consequential adhesions caused.

In a preliminary publication reporting outcomes in women undergoing laparoscopic UGOWR for recurrence of BOT taken from our own data, six patients underwent the procedure for management of a first recurrence of sBOT [16]. Table 7 demonstrates the updated findings, in which there have been no adverse surgical outcomes since.

The authors of this study have previously recommended the use of this novel technique for the management of second or third recurrences of disease, but only in women considered at high risk of declining ovarian reserve following surgery, whereby reproductive potential is limited and of high priority for the patient [16]. However, when used as the primary treatment for the initial management of BOT, the procedure has been successfully implemented, in which no recurrences have yet been reported. When UGOWR was performed in addition to other procedures considered FSS for the initial management of disease, there was one recurrence of sBOT. Furthermore, in six women who were treated for a first recurrence of BOT by UGOWR, 50% developed a second recurrence, which has either been managed conservatively, or with further UGOWR. It can be argued that the benefits of enabling the selected women to achieve pregnancy may considerably outweigh the risks of disease recurrence, in particular given the findings that recurrences of BOT in FSS are not malignant and can be detected early with surveillance ultrasound monitoring. When considering this treatment option for multiple recurrences of disease, it is at the clinicians’ discretion and responsibility to ensure the woman is fully informed regarding her choices, in particular, considering the number of surgical procedures performed is negatively associated with pregnancy outcome (*p* < 0.001) [2]. Although we appreciate the numbers presented in this study are too small to deduce significant conclusions, positive outcomes have been established from our data. However, further prospective studies are required to determine whether this adjunct to FSS impacts overall ovarian reserve and subsequent fertility outcomes in women with BOTs.

### 4.2. Reproductive Outcomes

Approximately 19.2% (*n* = 33) of women attempted pregnancy in our cohort. However, the numbers are too small to deduce whether pregnancy outcome is determined by the type of FSS. Previous studies have also provided inconclusive findings. For example, the cumulative pregnancy rate was similar when comparing USO and ovarian cystectomy (45.4% vs. 40.3%, respectively) [34], whereas multivariate analysis has shown no association between the conception and the type of surgery amongst 252 women in a separate study [2].

The majority of pregnancies achieved were spontaneous (78.9%), slightly lower than 93.4% reported from a study of 212 attempts [2]. Nonetheless, the findings suggest that FSS may preserve a significant quantity of healthy ovarian tissue, enabling women to achieve pregnancies without the need for assisted conception. This is exemplified by the fact that 50% of women who achieved pregnancy (*n* = 14), were able to conceive more than once postoperatively, thus fulfilling the purpose of FSS.

The pregnancy rate amongst the three histological subgroups ranged between 26–43%, consistent with previous reports [23,37]. However, not all studies within the literature that report a pregnancy rate were considered for the bias of their population sample. For example, a number of studies may not consider the proportion of women who wish to conceive or the previous history of infertility or account for the influence of histological or staging of disease or the age of women with fertility outcomes [38]. Furthermore, there are various predictors of pregnancy success, such as history of previous successful pregnancy (*p* = 0.005) and reduced number of surgeries performed that should be taken into consideration [2].

### 4.3. Strengths and Limitations

The strengths of this study include the duration of the long-term follow up and the sample size of the cohort included. Limitations, however, are certain findings that are reflective of a single centre and therefore subject to selection bias. In addition, retrospective analysis of data with a broad inclusion criteria, such as all women with BOT, are unlikely to provide novel findings with analysis of data from unequal sample sizes. The authors also acknowledge that certain clinical information was not adequately documented. It would therefore be beneficial in the future to carry out prospective studies. In such instances, a multi-centre collaboration may yield more influential findings.

## 5. Conclusions

The findings from this study suggest that non-FSS is associated with negative oncological outcomes when compared to FSS, as evidenced by the higher rate of recurrences of LGSC. This may be attributed to the stringent long-term follow-up and regular ultrasound surveillance that all FSS patients have in our centralised specialist clinic. This, in addition to the introduction of laparoscopic UGOWR, as a novel method of fertility preserving surgery, enables earlier detection and treatment of disease recurrence whilst preserving fertility. Furthermore, FSS has successfully enabled women attempting pregnancy to achieve spontaneous conceptions following management of disease.

## Figures and Tables

**Table 1:** Ultrasonographic features at diagnosis.

Feature	FSS *n* (%)	Non-FSS *n* (%)
Unilocular solid	14 (15.6%)	21 (25.6%)
Unilocular	3	2
	(3.3%)	(2.4%)
Multilocular	17 (18.9%)	14 (17.1%)
Other features	2 (2.2%)	2 (2.4%)
Data unattainable	54 (60.0%)	43 (52.4%)
Median maximum diameter (mm) (IQR)	115 (104.0)	110 (104.5)

Abbreviations: Interquartile range (IQR); Fertility Sparing Surgery (FSS); Number (*n*).

**Table 2:** Histopathological features from primary surgery.

Feature	FSS *n* (%)	Non-FSS *n* (%)
Other histological feature	16 (17.8%)	26 (31.7%)
Microinvasion	9 (10.0%)	8 (9.8%)
Micro-papillary pattern	14 (15.6%)	14 (17.1%)
Implants	8 (8.9%)	11 (13.4%)
Data unattainable	43 (47.8%)	23 (28.0%)
**Staging**		
1	69 (76.7%)	60 (73.1%)
2	4 (4.4%)	5 (6.1%)
3	5 (5.5%)	14 (17.1%)
4	1 (1.1%)	1 (1.2%)
Data unattainable	11 (12.2%)	2 (2.4%)

Abbreviations: Fertility Sparing Surgery (FSS); Number (*n*).

**Table 3:** Surgical management of primary Borderline Ovarian Tumour.

Surgery *n* (%)	Serous (*n* = 120)	Mucinous (*n* = 43)	Sero-Mucinous (*n* = 9)	*p* Value
Laparotomy	88 (73.3%)	34 (79.1%)	6 (66.6%)	0.689
Laparoscopy	32 (26.7%)	9 (20.9%)	3 (33.3%)	0.737
Fertility-Sparing Surgery	54 (45.0%)	30 (69.8%)	6 (66.7%)	0.666
Complete Debulking	66 (55.0%)	13 (30.2%)	3 (33.3%)	0.009 *
Disease Removed Intact	117 (97.5%)	41 (95.3%)	9 (100%)	0.013 *
Residual Disease	3 (2.5%)	2 (4.7%)	0	0.000 *
**Fertility-Sparing Surgery**				
Unilateral Salpingo-oophorectomy	11 (9.2%)	5 (11.6%)	4 (44.4%)	0.001 *
Ovarian Cystectomy	24 (20.0%)	9 (20.9%)	0	0.001 *
Ultrasound-guided ovarian wedge resection	3 (2.5%)	0	1 (11.1%)	0.203

* Independent samples Kruskal-Wallis Test (* *p* value significant when <0.05).

**Table 4:** Recurrence rate of Borderline Ovarian Tumour following surgical management of primary disease.

Type of Surgery	Total Number Performed	Number of Recurrences	Recurrence Rate (%)
Unilateral salpingo-oophorectomy	20	6	30.0
Ovarian cystectomy	33	10	30.3
Ultrasound-guided ovarian wedge resection	4	0	0
Other fertility-sparing surgical procedure (including combined procedures)	33	7	21.2
All fertility-sparing surgery	90	23	25.6
Complete debulking	78	6	7.7
Not documented	4	0	0

**Table 5:** Comparisons between types of fertility-sparing surgery and recurrence of Borderline Ovarian Tumour.

Fertility Sparing Surgery	X^2^	*p* Value
Unilateral salpingo-oophorectomy v. Ovarian cystectomy	2.93	0.182
Unilateral salpingo-oophorectomy v. Ultrasound-guided ovarian wedge resection	Non calculable	Non calculable
Ultrasound-guided ovarian wedge resection v. Ovarian cystectomy	Non calculable	Non calculable
Fertility-sparing surgery v. non-fertility-sparing surgery	20.61	0.000 *

* Pearson Chi-Squared Statistical Test of Association (* *p* value significant when <0.05).

**Table 6:** Comparison amongst women with non-invasive recurrences and malignant transformation.

Variable	X^2^	*p* Value
Type of surgery (e.g., Fertility Sparing Surgery or Non-Fertility Sparing Surgery)	9.9	0.002 *
Age at diagnosis	7.0	0.99
Histological subtype at diagnosis	21.5	0.000 *
		
Staging of disease at diagnosis	8.0	0.87

* Pearson Chi-Squared Statistical Test of Association (* *p* value significant when <0.05).

**Table 7:** Post-operative reproductive outcomes in women with Borderline Ovarian Tumour.

Reproductive Outcome	Serous (*n* = 120)	Mucinous (*n* = 43)	Sero-Mucinous (*n* = 9)	Total (*n*)
**Total number of pregnancies (*n*)**				
Spontaneous and IVF	37	15	3	55
**Spontaneous pregnancies only *n* (%)**				
Total number of women of reproductive age achieving pregnancy	15/68 (22.1%)	6/31 (19.4%)	2/7 (28.6%)	23/106 (21.6%)
Total number of spontaneous conceptions by women achieving spontaneous pregnancy (*n*)	30	11	2	43
**Outcome of spontaneous pregnancies (per conception) *n* (%)**				
Miscarriage	7 (23.3%)	5 (45.4%)	0	12/43 (27.9%)
Successful livebirth	23 (76.7%)	6 (54.5%)	2 (100%)	31/43 (72.1%)
**IVF pregnancies only *n* (%)**				
Number of women attempting IVF	7/68 (10.3%)	2/31 (6.5%)	1/7 (14.3%)	10/106 (9.4%)
Number of cycles	7	4	1	12
**Outcome of IVF pregnancies (per cycle) *n* (%)**				
Miscarriage	1 (14.3%)	2 (50.0%)	1 (100%)	4/12 (33.3%)
Successful livebirth	5 (71.4%)	1 (25.0%)	0	6/12 (50.0%)
Unsuccessful implantation	1 (14.3%)	0	0	1/12 (8.3%)
Ectopic pregnancy	0	1 (25.0%)	0	1/12 (8.3%)

Abbreviations: In Vitro fertilization (IVF); Fertility Sparing Surgery (FSS); Number (*n*).

**Table 8:** Surgical and reproductive outcomes following laparoscopic ultrasound-guided ovarian wedge resection.

Patient	Age	Gravida	Parity	Initial Surgery	1st Recurrence	2nd Recurrence	3rd Recurrence	Attempt of Pregnancy after FSS	Cryopreserved Oocytes or Embryo Post-FSS	No. of Pregnancies after FSS	Pregnancy Outcome after FSS
**Serous**											
1	38	0	0	UGOWR	-	-	-	No	No	0	-
2	22	0	0	UGOWR	-	-	-	No	No	0	-
3	29	0	0	UGOWR	-	-	-	No	No	0	-
4	30	0	0	USO and contralateral UGOWR	USO and total Infra-colic Omenectomy	-	-	No	Yes	0	-
5	23	0	0	Infra-colectomy, ablation of pelvic deposits & UGOWR	-	-	-	No	Yes	0	-
6	33	3	2	USO	UGOWR	Conservative	-	Yes	No	1 (Spontaneous)	Miscarriage
7	23	0	0	USO	UGOWR	-	-	Yes	Yes	1 (IVF Twins)	Livebirth
8	35	1	1	Ovarian Cystectomy	UGOWR	UGOWR	UGOWR	Yes	Yes	1 (Spontaneous)	Livebirth
9	31	0	0	Partial Oophorectomy	UGOWR	Conservative	-	Yes	Yes	0	-
10	33	0	0	Ovarian Cystectomy	UGOWR	-	-	No	Yes	0	-
11	22	0	0	USO and contralateral ovarian biopsy	UGOWR	-	-	No	Yes	0	-
**Sero-Mucinous**											
12	26	0	0	UGOWR	-	-	-	No	No	0	-

**Abbreviations:** Fertility Sparing Surgery (FSS); Ultrasound guided ovarian wedge resection (UGOWR); Unilateral Salpingo oophorectomy (USO); In Vitro fertilisation (IVF).

## Data Availability

The data presented in this study are available on request from the corresponding author.

## Supplementary Materials

The following supporting information can be downloaded at: https://www.mdpi.com/article/10.3390/cancers14061485/s1, Table S1: Surgical management of primary Borderline Ovarian Tumour.
