# Supplementary material for: Fertility Sparing Surgery and Borderline Ovarian Tumours
Source: Cancers (Basel). 2022 Mar 14;14(6):1485. doi: 10.3390/cancers14061485 (PMC8946233; doi:10.3390/cancers14061485)
Supplement: Supplementary file 1 [file cancers-14-01485-s001.zip › cancers-1552880-supplementary.pdf]

cancers MDPI

Supplementary Materials

Fertility Sparing Surgery and Borderline Ovarian Tumours

Lorraine S. Kasaven [1,2,*], Mehar Chawla [3], Benjamin P. Jones [3], Maya Al-Memar [4], Nicolas Galazis [5], Yousra Ahmed-Salim [5], Mona El-Bahrawy [6], Stuart Lavery [7], Srdjan Saso [8] and Joseph Yazbek [8]

1 Department of Cancer and Surgery, Imperial College London, South Kensington Campus, London SW7 2AZ, UK
2 Department of Cutrale Perioperative & Ageing Group, Imperial College London, South Kensington Campus, London SW7 2AZ, UK
3 Department of Obstetrics and Gynaecology, West Middlesex University Hospital, Chelsea and Westminister NHS Foundation Trust, London TW7 6AF, UK; mehar.chawla@nhs.net, benjamin.jones@nhs.net
4 Department of Obstetrics and Gynaecology, Queen Charlotte's and Chelsea Hospital, Imperial College NHS Trust, London W12 0HS, UK; maya.almemar@nhs.net
5 Department of Obstetrics and Gynaecology, Imperial College NHS Trust, London W12 0HS, UK; nicolas.galazis@nhs.net; Yousra.ahmed-salim@nhs.net
6 Department of Metabolism, Digestion and Reproduction, Imperial College London, Hammersmith Hospital, London W12 0HS, UK; m.elbahrawy@imperial.ac.uk
7 Department of Reproductive Medicine, Hammersmith Hospital, Imperial College NHS Trust, London W12 0HS, UK; stuart.lavery@nhs.net
8 Department of Gynaecological Oncology, Queen Charlotte's and Chelsea Hospital, Imperial College NHS Trust, London W12 0HS, UK; srdjan.saso01@imperial.ac.uk; joseph.yazbek@nhs.net
* Correspondence: l.kasaven@nhs.net

Table S1. Surgical management of primary Borderline Ovarian Tumour.

Other Types of Fertility Sparing Surgical Procedures (Combined Procedures)				
**Type of Fertility Sparing Surgery**	**Serous (n = 120)**	**Mucinous (n = 43)**	**Sero-Mucinous (n = 9)**	***p* Value**
Unilateral Salpingo-oophorectomy and Contralateral Ovarian Cystectomy	4 (3.3%)	0	0	Non calculable
Unilateral Salpingo-oophorectomy and Omenectomy	0	2 (4.7%)	0	Non calculable
Unilateral Salpingo-oophorectomy and Appendicectomy	0	9 (20.9%)	0	Non calculable
Ovarian Cystectomy, Omental Biopsy and Resection of Implant	1 (0.8%)	0	0	Non calculable
Unilateral Salpingo-oophorectomy and Partial Oophorectomy	1 (0.8%)	0	0	Non calculable
Ovarian Cystectomy and Biopsy of Ipsilateral Ovary	1 (0.8%)	0	1 (11.1%)	Non calculable
Unilateral Salpingo-oophorectomy, Peritoneal Stripping and Omental Biopsy	1 (0.8%)	0	0	Non calculable
Unilateral Salpingo-oophorectomy and Contralateral Ultrasound guided ovarian wedge resection	1 (0.8%)	0	0	Non calculable
Unilateral Salpingo-oophorectomy, Omental Biopsy and Contralateral Ovarian Biopsy	1 (0.8%)	0	0	Non calculable
Ultrasound guided ovarian wedge resection, Ureterolysis, Pelvic Peritonectomy and Infra-colic Omenectomy	1 (0.8%)	0	0	Non calculable
Unilateral Salpingo-oophorectomy, Appendicectomy, Omenectomy, Pelvic Lymph Node Resection	1 (0.8%)	0	0	Non calculable
Unilateral Salpingo-oophorectomy, Appendicectomy, Infra-colic Omenectomy and Peritonectomy	0	2 (4.7%)	0	Non calculable
Bilateral salpingo-oophorectomy	1 (0.8%)	0	0	Non calculable
Bilateral Ovarian Cystectomy and Omental Biopsy	1 (0.8%)	0	0	Non calculable
Salpingectomy	1 (0.8%)	1 (2.3%)	0	Non calculable
Ovarian Cystectomy and Appendicectomy	0	1 (2.3%)	0	Non calculable
Ovarian Cyst Deroofing and Drainage	1 (0.8%)	0	0	Non calculable
Partial Oophorectomy, Appendicectomy and biopsy	0	1 (2.3%)	0	Non calculable

Abbreviations: Number (n).
